# An Enhanced Whale Optimization Algorithm with outpost and multi-population mechanisms for high-dimensional optimization and medical diagnosis

**DOI:** 10.1371/journal.pone.0325272

**Published:** 2025-06-03

**Authors:** Kankan Tang, Lin Zhang

**Affiliations:** Department of Respiratory and Critical Care Medicine, the First Medical Center, Chinese PLA General Hospital, Beijing, Haidian District, China; Central University of Haryana School of Engineering and Technology, INDIA

## Abstract

Swarm intelligence optimization algorithms represent a significant branch of nature-inspired computational methods, designed to solve complex optimization problems by simulating the collective behavior of biological systems. Whale optimization algorithm (WOA) is a newly developed meta-heuristic algorithm, which is mainly based on the predation behavior of humpback whales in the ocean. This study proposes an enhanced version of the WOA, named the Outpost-based Multi-population Whale Optimization Algorithm (OMWOA), which integrates two key mechanisms: the outpost mechanism and a multi-population enhanced mechanism. These modifications aim to improve the algorithm’s performance in terms of solution accuracy and convergence rate. The effectiveness of OMWOA is thoroughly evaluated by benchmarking it against state-of-the-art evolutionary algorithms from the IEEE CEC 2017 and IEEE CEC 2022 competitions. Additionally, this study provides a detailed analysis of the influence of the outpost and multi-population mechanisms on OMWOA’s performance, as well as its scalability in problems of varying dimensionalities. To validate its applicability in real-world problems, the proposed algorithm is combined with Kernel Extreme Learning Machine (KELM) for solving medical disease diagnosis tasks. The experimental results demonstrate the superior performance of OMWOA in terms of diagnostic accuracy across five medical datasets, highlighting its potential for real-world applications.

## 1. Introduction

Swarm intelligence optimization algorithms represent a significant branch of nature-inspired computational methods, designed to solve complex optimization problems by simulating the collective behavior of biological systems. These algorithms draw inspiration from the self-organizing and cooperative behaviors observed in nature, such as the foraging of ant colonies, the synchronized movement of bird flocks, and the coordinated hunting of wolves. By emulating these behaviors, swarm intelligence algorithms rely on the interaction of simple agents operating under decentralized control. Each agent follows basic rules, yet their collective interactions lead to emergent global intelligence capable of efficiently exploring and exploiting the search space. Key characteristics of swarm intelligence algorithms include adaptability to changing environments, scalability for handling high-dimensional problems, and the ability to avoid local optima by maintaining a balance between exploration and exploitation.

Swarm intelligence and evolutionary optimization techniques are primarily inspired by natural processes such as evolution, hunting, foraging, and survival strategies observed in groups or individuals in nature [1–3]. In recent years, these optimization methods have become crucial in addressing numerous large-scale and real-world challenges [4–6]. Compared to gradient-based methods, swarm intelligence algorithms have demonstrated superior efficiency in solving complex optimization tasks [7]. Notable examples include traditional approaches such as particle swarm optimization (PSO) [8,9] and ant colony optimization (ACO) [10,11]. Recently developed algorithms include the artificial bee colony algorithm (ABC) [12], multi-verse optimizer (MVO) [13], fruit fly optimization algorithm (FOA) [14,15], grasshopper optimization algorithm (GOA) [16], bat algorithm (BA) [17,18], chicken swarm optimization (CSO) [19], and artificial fish swarm algorithm (AFSA) [20]. The whale optimization algorithm (WOA), introduced by Mirjalili in 2016 [21], emulates the hunting behavior of whales during foraging to efficiently explore and exploit potential optimal or near-optimal solutions.

In recent years, the Whale Optimization Algorithm (WOA) has been extensively applied across various domains, including feature selection [22,23], retinal vascular recognition [24], neural network optimization [25,26], image segmentation [27], image retrieval [28], key recognition [29], wind speed prediction [30], and sentiment analysis [31]. WOA is characterized by its simplicity and robust global search capabilities, which allow it to outperform algorithms like PSO and SCA in terms of solution quality. However, WOA faces challenges when dealing with complex and high-dimensional problems, particularly in terms of a slow convergence rate and suboptimal solution quality during the latter stages of iteration. To address these limitations, researchers have proposed numerous enhancements. Zhou *et al.* [32] introduced a Lévy flight-based WOA (LWOA) for engineering optimization, leveraging Lévy flight trajectories to enhance population diversity and mitigate premature convergence, thus improving the ability to escape local optima. Mafarja *et al.* [22] developed a binary version of WOA that integrates evolutionary operators such as selection, crossover, and mutation. Yousri *et al.* [33] explored the use of ten different chaotic maps to optimize WOA’s parameters, resulting in improved performance. Moreover, variants like CWOA and adaptations of standard WOA have been utilized to estimate chaotic behavior parameters in PMSM under noise-free and noisy conditions, achieving lower error rates, faster convergence, and reduced execution times. Bhowmik *et al.* [34] proposed balancing local and global searches by employing non-linear and random variations of parameter “a” and an inertial weight strategy for updating parameter “c.” Sun *et al.* [25] introduced chaos into WOA’s initialization process, using chaotic dynamics to enhance search diversity and reduce self-centered tendencies in the search process. Yaqoob *et al.* [35] proposed a novel method called the Harris Hawks Optimization and Cuckoo Search Algorithm (HHOCSA), which is applicable to commonly used machine learning classifiers. Alnowibet *et al.* [36] proposed two major improvements in the WOA: first, a reverse learning-based method was employed during the initialization phase, and second, a Cauchy mutation operator was introduced during the position update phase. The proposed variant is named the Enhanced Whale Optimization Algorithm (AWOA).

In this article, in order to improve the performance of WOA, we incorporate an outpost mechanism and a multi-population enhanced mechanism into WOA. Incorporating an outpost mechanism and a multi-population enhanced mechanism into the Whale Optimization Algorithm (WOA) can significantly improve its search efficiency and solution quality, especially when dealing with complex and high-dimensional optimization problems. The outpost mechanism helps in maintaining a set of “outpost” individuals, which are strategically distributed across the search space to act as exploratory agents. These outposts are designed to remain in diverse areas of the solution space, facilitating a more extensive and diversified exploration process. By doing so, they reduce the likelihood of the algorithm getting trapped in local optima, thereby improving the global search capabilities of the WOA. These outposts serve as anchor points, guiding the search towards promising regions while maintaining a high level of exploration.

On the other hand, the multi-population enhanced mechanism enhances the balance between exploration and exploitation by employing multiple sub-populations that operate concurrently within the search space. Each sub-population independently explores different regions, which leads to a broader and more thorough search. This mechanism enables the algorithm to avoid the common pitfall of converging to a single, potentially suboptimal solution. The diversity introduced by multiple populations allows the algorithm to maintain a higher degree of flexibility, ensuring that different regions of the search space are explored in parallel, thereby increasing the likelihood of finding the global optimum.

When combined, these two mechanisms provide a powerful strategy for enhancing the WOA’s performance. The outpost mechanism ensures the algorithm explores various regions of the solution space without premature convergence, while the multi-population mechanism enables the algorithm to explore multiple areas simultaneously, reducing the risk of stagnation. Together, these mechanisms promote a more balanced and effective exploration-exploitation trade-off, leading to improved convergence rates, higher quality solutions, and greater robustness in solving complex optimization problems. These enhancements allow the WOA to outperform traditional single-population algorithms, making it a more reliable and efficient tool for tackling large-scale, high-dimensional optimization tasks.

In summary, the main contributions of this study are outlined as follows:

1. This study introduces an enhanced version of WOA, referred to as OMWOA, which incorporates the outpost mechanism and a multi-population enhanced mechanism.
2. The performance of the proposed OMWOA was assessed by comparing it with leading evolutionary algorithms from the IEEE CEC 2017 and IEEE CEC 2022 competitions. This study further provides a comprehensive analysis of the impact of the two enhancement mechanisms on OMWOA’s performance, as well as an evaluation of its scalability across different dimensionalities.
3. To test the optimization performance of OMWOA in real-world problems, we combined OMWOA with KELM to solve the diagnosis problem of medical diseases. OMWOA has achieved good diagnostic results on 5 medical datasets.

This paper is organized as follows. Section 2 briefly describes WOA. The proposed OMWOA is described in detail in Section 3. Section 4 introduces and analyses OMWOA in benchmark function testing. The Section 5 analyzes the experimental results of medical problems. The Section 6 summarizes the whole paper and looks forward to the future.

## 2. An overview of WOA

WOA, proposed by Mirjalili in 2016 [21], is a meta-heuristic algorithm inspired by the hunting behavior of humpback whales. Humpback whales employ a distinctive hunting strategy: they dive underwater, spiral upwards from depths of about 12 meters, and emit bubbles of varying sizes. These bubbles rise to the water’s surface in unison, forming a spiral bubble net that ensnares and directs prey towards the center. With its mouth nearly vertical amidst the bubble circle, the whale engulfs the trapped prey. WOA seeks to emulate the spiral bubble net tactic employed by humpback whales, accomplishing foraging through three mechanisms: spiral predation, random predation and encirclement, and contraction. The mathematical model of WOA is expounded in the ensuing sections.

### 2.1 Encircling prey

In the wild, whales possess the ability to pinpoint the whereabouts of their prey and encircle them for predation. WOA operates under the assumption that the optimal position within the existing population represents the prey; all remaining whale individuals converge around this prey, with their locations updated according to Eq. (1) and Eq. (2).


$$D=\left|CX^{*}(t)-X(t)\right|$$ (1)

$$X(t+1)=X^{*}(t)-A\times D$$ (2)

In this equation, *t* denotes the iteration count, while *A* and *C* denote coefficient vectors. $X^{*}(t)$ represents the best position within the current population, with *A* and *C* derived from Eq. (3) and Eq. (4).


$$A=2ar_{1}-a$$ (3)

$$C=2r_{2}$$ (4)

Among these parameters, *r*_1_ and *r*_2_ are random numbers within the interval (0,1). The value of *a* linearly decreases from 2 to 0, where *t* represents the current iteration number and $T_{\max}$ is the maximum iteration number.

### 2.2 Spiral bubble-net feeding maneuver

During hunting, humpback whales ascend in spirals towards their prey. In WOA, whales utilize Eq. (5) to adjust their position as they swim towards the optimal individual.


$$X(t+1)=X^{*}(t)+D_{p}e^{bl}cos(2\pi l)$$ (5)

In this context, $D_{p}=|X^{*}(t)-X(t)|$ signifies the distance between the optimal individual *X* before the update and the optimal position $X_{best}$. *b* serves as a constant that defines the spiral shape, while *l* represents a random number within the range of [−1,1]. In the mathematical model, given that the spiral predation of wha*l*es involves both movement around the outer ring and contraction of the enclosure, half of the probability will opt for the contraction mechanism, as exemplified by Eq. (2), to update the whales’ locations.

### 2.3 Searching for prey

The whale’s search and predatory actions occur randomly based on its position. Within the WOA framework, the whale adjusts its position using Eq. (6) and Eq. (7).


$$D=CX_{rand}-X(t)$$ (6)

$$X(t+1)=X_{rand}-A\times D$$ (7)

In this context, $X_{rand}$ represents a randomly chosen whale position vector.

The pseudocode of WOA is shown as in Algorithm 1.

**Algorithm** 1. Pseudocode of WOA

Begin by initializing a set of agents *X*_*i*_ (*i* = 1,2,3...., *n*) with random distribution.

Evaluate the fitness of each search agent.

Identify the optimal search agent X* among them.

While (*FEs* < MaxFEs)

 for each search agent

  Update *a, A, C, L*, and *p*

   if(*p* < 0.5)

    if(|*A*| < 1)

     Update the position of the search agents.

    else if(|*A*| > 1)

     Randomly select a search agent $X_{rand}$.

     Update the position of the search agent.

    end if

   else if(*p* > 0.5)

     Position has been refreshed using the spiral Eq. (5).

   end if

 end for

 Verify whether any search agent exceeds the search space boundaries and rectify it accordingly.

 Evaluate the fitness of the solutions obtained.

 Adjust X* if an improved solution is detected by the method.

 *t* = *t* + 1

end while

return x*

## 3. The proposed OMWOA

In this section, we provide a detailed description of OMWOA. Unlike the original algorithm, OMWOA incorporates two additional strategies. Firstly, it integrates outpost mechanism, enhancing the basic algorithm. Additionally, a multi-population enhanced mechanism is introduced to improve the convergence speed and enhance the solution quality of the algorithm.

### 3.1 Outpost mechanism

Initially, the population’s fitness value is compared with the value obtained in the previous iteration. If the fitness value from the current iteration is better, the position is moved to the current one. If the fitness value does not improve, the position remains in the sub-optimal one.


$$\left\{ \begin{array}{c} {}[\lambda ]=\min\left(function(S_{temp}),function(S_{i})\right)\\ S_{i}=S_{\lambda } \end{array}\right.\;$$ (8)

λ signifies the position’s location in this case. The updated population position will subsequently take the place of the current population position, as indicated by the formula above.

In the second phase, the individual investigates in a random direction and distance around the optimal value. The position distribution for this random search can be approximated by a Gaussian distribution. The formula for the probability density function of the Gaussian distribution is described in Eq. (9).


$$f(x)=\frac{1}{\sigma \sqrt{2\pi }}e^{\frac{(x-\mu {)}^{2}}{2\sigma^{2}}},\;-\infty <x<\infty$$ (9)

In this situation, $\sigma^{2}$ is the standard deviation among the individuals, and μ represents the mean value of the entire population. The normal distribution characteristics allow us to derive the individuals’ distribution density. Therefore, in this mechanism, a normal distribution with μ=0,σ=1 is utilized for all problems. The variables generated in this way are employed in this study as outlined below:


$${Mut}_{i}^{d}=X_{i}+X_{i}⊕G(\vartheta )$$ (10)

G(ϑ) refers to a normal distribution that generates a Gaussian gradient vector, and ⊕ denotes the dot product (entry-wise multiplication). In the third step, we use the Eq. (11) to illustrate an individual’s inclination when updating.


$$\left\{ \begin{array}{c} X_{axis}=X_{axis}\pm X_{bestindex}\\ Y_{axis}=Y_{axis}\pm Y_{bestindex} \end{array}\right.\;$$ (11)

In Eq. (11), if the fitness value discovered during the current iteration exceeds the current fitness value, the formula employs the plus sign to update the current best position and the subgroup’s optimal fitness value. Otherwise, the formula uses the minus sign.

**3.2 The multi-population enhanced mechanism**

In the original algorithm, once a particular individual discovers the optimal solution, other individuals align in the optimal direction, causing a decline in diversity. To enhance the global optimization ability, especially for multi-modal challenges, a multi-population mechanism was introduced into FOA. This mechanism comprises two parameters, α and Ω.


α=2(1−FEs/MaxFEs) (12)

Ω=rand(LB,UB) (13)

In this scenario, *FEs* indicates the current evaluation count, while *MaxFEs* signifies the maximum number of evaluations permitted. LB and UB correspond to the lower and upper bounds of the problem, respectively.

The population is split into *M* subgroups, each independently searching. Meanwhile, some individuals in each subgroup have a probability of engaging in a global search, and the search radius reduces with increasing iterations. The position of the individual is explicitly depicted in Eq. (14).


$$S_{i}=\left\{ \begin{array}{c} S_{i}+sign(rand-0.5)\times \alpha \times \Omega ,\;i=ceil(rand(popsize))\\ S_{i},\;others \end{array}\;\right.\;$$ (14)

Where $i,i\in N^{+}$ denotes the individual that has undergone mutation.

The pseudocode of OMWOA is shown in Algorithm 2. The OMWOA is a metaheuristic approach designed for global optimization tasks. Initially, a set of search agents *X*_*i*_ is initialized with random distributions. Each agent’s fitness is evaluated, and the optimal agent X* is identified. The algorithm iterates until a maximum number of function evaluations *FEs* is reached. During each iteration, parameters *a, A, C, L*, and *p* are updated, influencing agent movements based on predefined conditions. Depending on the value of p, agents either update their positions using spiral equations or interact with randomly selected agents. Post-update, mechanisms like outpost and multi-population enhancements foster diversity and improve search effectiveness. Boundary checks ensure agents remain within the search space. The process continually evaluates solution fitness, updating X* if better solutions are found. OMWOA’s iterative approach aims to converge towards optimal solutions efficiently across various optimization challenges.

**Algorithm** 2. Pseudocode of OMWOA

Begin by initializing a set of agents *X*_*i*_ (*i* = 1,2,3...., *n*) with random distribution.

Evaluate the fitness of each search agent.

Identify the optimal search agent X* among them.

While (*Fes *< *MaxFEs*)

 for each search agent

  Update *a, A, C, L*, and *p*

   if(*p* < 0.5)

    if(|*A*| < 1)

     Update the position of the search agents.

    else if(|*A*| > 1)

     Randomly select a search agent $X_{rand}$.

     Update the position of the search agent.

    end if

   else if(*p* > 0.5)

     Position has been refreshed using the spiral Eq. (5).

 end if

 end for

 Updating X by outpost mechanism;

 Updating X by multi-population enhanced mechanism;

 Verify whether any search agent exceeds the search space boundaries and rectify it accordingly.

 Evaluate the fitness of the solutions obtained.

 Adjust X* if an improved solution is detected by the method.

 *t* = *t* + 1

end while

return X*

## 4. Experimental results and discussions

Within this section, we commence with a presentation of comparative results and proceed to discuss our observations in detail. We begin by analyzing algorithm parameters, followed by conducting simulation experiments on benchmark functions to comprehensively validate the effectiveness of the OMWOA. Lastly, we explore practical applications of the algorithm.

### 4.1 Benchmark functions

#### 4.1.1IEEE CEC 2017 benchmark functions.

Table 1 shows the details of the IEEE CEC 2017 benchmark functions. For unbiased outcomes, all algorithms were tested under consistent conditions: population size and maximum evaluation times were set to 30 and 300,000, respectively. Unless otherwise specified, the population size was set to 30, which is a commonly used setting in the field of evolutionary computation. A population size of 30 is sufficient to ensure adequate exploration of the search space. For the total number of iterations, we set it to 300,000, corresponding to a problem dimension of 30 and 10,000 iterations per dimension. This setting is widely adopted in evolutionary computation to facilitate fair performance comparisons between algorithms. In the scalability experiments, we further validated our algorithm across different dimensions, including 30, 50, and 100, to demonstrate its performance under varying problem scales. Each algorithm underwent independent testing 30 times per benchmark function. Conducting 30 independent runs helps to reduce the influence of randomness and enhances the credibility of the experimental results. The Friedman test, a non-parametric statistical method for comparative analysis, was employed to evaluate and rank algorithm performance across the benchmark functions. The average ranking value (ARV) derived from the Friedman test allows for further statistical comparisons, where algorithms with lower ARV demonstrate better performance. The Friedman test allows for a more detailed and intuitive analysis of performance differences among algorithms. By setting the confidence level at 0.05, our experiments demonstrated that the proposed OMWOA algorithm consistently outperformed the compared algorithms across 30 independent runs.

**Table 1:** IEEE CEC 2017 benchmark function specifications.

Function Equation	Dim	Optimum
$f_{1}(x)=x_{1}^{2}+10^{6}\sum_{i=2}^{D}x_{i}^{2}$	30	100
$f_{2}(x)=\sum_{i=1}^{D}\left|x_{i}^{2}\right|$	30	200
$f_{3}(x)=\sum_{i=1}^{D}x_{i}^{2}+{\left(\sum_{i=1}^{D}0.5x_{i}^{2}\right)}^{2}+{\left(\sum_{i=1}^{D}0.5x_{i}^{2}\right)}^{4}$	30	300
$f_{4}(x)=\sum_{i=1}^{D-1}\left(100{\left(x_{i}^{2}-x_{i+1}\right)}^{2}+{\left(x_{i}-1\right)}^{2}\right)$	30	400
$f_{5}(x)=\sum_{i=1}^{D}\left(x_{i}^{2}-10\cos\left(2\pi x_{i}\right)+10\right)$	30	500
$f_{6}(x)=g\left(x_{1},x_{2}\right)+g\left(x_{2},x_{3}\right)+\ldots +g\left(x_{\text{D-}1},x_{D}\right)+g\left(x_{D},x_{1}\right)$ $g(x,y)=0.5+\frac{\left(\sin^{2}\left(\sqrt{x^{2}+y^{2}}\right)-0.5\right)}{{\left(1+0.001\left(x^{2}+y^{2}\right)\right)}^{2}}$	30	600
$f_{7}(x)=\min\left({\sum_{i=1}^{D}\left({\overset{̑}{x}}_{i}-\mu_{0}\right)}^{2},dD+{s\sum_{i=1}^{D}\left({\overset{̑}{x}}_{i}-\mu_{1}\right)}^{2}\right)+10\left(D-\sum_{i=1}^{D}\cos\left(2\pi {\overset{̑}{z}}_{i}\right)\right)$	30	700
$f_{8}(x)=\sum_{i=1}^{D}\left(z_{i}^{2}-10\cos\left(2\pi z_{i}\right)+10\right)+{f_{13}}^{*}$	30	800
$f_{9}(x)=\sin^{2}\left(\pi w_{1}\right)+\sum_{i=1}^{D}{\left(w_{i}-1\right)}^{2}\left[1+10\sin^{2}\left(\pi w_{i}+1\right)\right]+{\left(w_{D}-1\right)}^{2}\left[1+\sin^{2}\left(2\pi w_{D}\right)\right]$	30	900
$f_{10}(x)=418.9829\times D-\sum_{i=1}^{D}g\left(z_{i}\right)\;,\;z_{i}=x_{i}+4.209687462275036e+002$	30	1000
$f_{11}(x)={\sum_{i=1}^{D}\left(10^{6}\right)}^{\frac{i-1}{D-1}}x_{i}^{2}$	3	1100
$f_{12}(x)=10^{6}x_{1}^{2}+\sum_{i=2}^{D}x_{i}^{2}$	3	1200
$f_{13}(x)=-20\exp\left(-0.2\sqrt{\frac{1}{D}\sum_{i=1}^{D}x_{i}^{2}}\right)-\exp\left(\frac{1}{D}\sum_{i=1}^{D}\cos\left(2\pi x_{i}\right)\right)+20+e$	3	1300
$f_{14}(x)=\sum_{i=1}^{D}\left(\sum_{k=0}^{kmax}\left[a^{k}\cos\left(2\pi b^{k}(x+0.5\right))\right]\right)-D\sum_{k=0}^{kmax}\left[a^{k}\cos\left(2\pi b^{k}\mathrm{.0}\mathrm{.5}\right)\right]$	4	1400
$f_{15}(x)=\sum_{i=1}^{D}\frac{x_{i}^{2}}{4000}-\prod_{i=1}^{D}\cos\left(\frac{x_{i}}{\sqrt{i}}\right)+1$	4	1500
$f_{16}(x)=\frac{10}{D^{2}}{\prod_{i=1}^{D}\left(1+i\sum_{j=1}^{32}\frac{\left|2^{j}x_{i}-round\left(2^{j}x_{i}\right)\right|}{2^{j}}\right)}^{\frac{10}{D^{1.2}}}-\frac{10}{D^{2}}$	4	1600
$f_{17}(x)={\left|\sum_{i=1}^{D}x_{i}^{2}-D\right|}^{1/4}+\left(0.5\sum_{i=1}^{D}x_{i}^{2}+\sum_{i=1}^{D}x_{i}\right)/D+0.5$	5	1700
$f_{18}(x)={\left|{\left(\sum_{i=1}^{D}x_{i}^{2}\right)}^{2}-{\left(\sum_{i=1}^{D}x_{i}\right)}^{2}\right|}^{1/4}+\left(0.5\sum_{i=1}^{D}x_{i}^{2}+\sum_{i=1}^{D}x_{i}\right)/D+0.5$	5	1800
$f_{19}(x)=f_{7}\left(f_{4}\left(x_{1},x_{2}\right)\right)+f_{7}\left(f_{4}\left(x_{2},x_{3}\right)\right)+\ldots +f_{7}\left(f_{4}\left(x_{D-1},x_{D}\right)\right)+f_{7}\left(f_{4}\left(x_{D},x_{1}\right)\right)$	5	1900
$f_{20}(x)={\left[\frac{1}{D-1}\sum_{i=1}^{D-1}\left(\sqrt{s_{i}}\cdot \left(\sin\left(50.0s_{i}^{0.2}\right)+1\right)\right)\right]}^{2},s_{i}=\sqrt{x_{i}^{2}+x_{i+1}^{2}}$	6	2000
$f_{21}(x)=f_{1}\left(M\left(x-o_{1}\right)\right)+{f_{21}}^{*}$	3	2100
$f_{22}(x)=f_{2}\left(M\left(x-o_{2}\right)\right)+{f_{22}}^{*}$	3	2200
$f_{23}(x)=f_{3}\left(M\left(x-o_{3}\right)\right)+{f_{23}}^{*}$	4	2300
$f_{24}(x)=f_{4}\left(M\left(\frac{2.048\left(x-o_{4}\right)}{100}\right)+1\right)+{f_{24}}^{*}$	4	2400
$f_{25}(x)=f_{5}\left(M\left(x-o_{5}\right)\right)+{f_{25}}^{*}$	5	2500
$f_{26}(x)=f_{20}\left(M\left(\frac{2.048\left(x-o_{6}\right)}{100}\right)\right)+{f_{26}}^{*}$	5	2600
$f_{27}(x)=f_{7}\left(M\left(\frac{600\left(x-o_{7}\right)}{100}\right)\right)+{f_{27}}^{*}$	6	2700
$f_{28}(x)=f_{8}\left(\frac{5.12\left(x-o_{8}\right)}{100}\right)+{f_{28}}^{*}$	6	2800
$f_{29}(x)=f_{9}\left(M\left(\frac{5.12\left(x-o_{9}\right)}{100}\right)\right)+{f_{29}}^{*}$	3	2900
$f_{30}(x)=f_{30}\left(M\left(\frac{1000\left(x-o_{10}\right)}{100}\right)\right)+{f_{30}}^{*}$	3	3000

#### 4.1.2 IEEE CEC 2022 benchmark functions.

Table 2 shows the details of the IEEE CEC 2022 benchmark functions.

**Table 2:** IEEE CEC 2022 benchmark function specifications.

Functions	Describe	$f_{i}$
F1	Shifted and full Rotated Zakharov	300
F2	Shifted and full Rotated Rosenbrock	400
F3	Shifted and full Rotated Expanded Schaffer’s f6	600
F4	Shifted and full Rotated Non-Continuous Restrain	800
F5	Shifted and full Rotated Levy	900
F6	Hybrid	1800
F7	Hybrid	2000
F8	Hybrid	2200
F9	Composition	2300
F10	Composition	2400
F11	Composition	2600
F12	Composition	2700

### 4.2 Ablation analysis

This section clarifies the improved effects of two enhancement mechanisms on OMWOA through ablative experiments, which are essential in scientific research. Ablative experiments are crucial for validating the robustness and reliability of research findings. By systematically removing a variable or factor and observing its impact, these experiments confirm the observed effects and eliminate other potential explanations. This methodology allows researchers to ascertain the contribution and significance of each factor in the study, thereby affirming the reliability of the results and mitigating potential confounding variables. Ablative experiments are indispensable for verifying scientific hypotheses, supporting research conclusions, and enhancing the credibility and reproducibility of research. Table 3 and Table 4 presents the experimental results, where OWOA represents WOA improved solely by the outpost mechanism, and MWOA denotes WOA improved exclusively by the multi-population enhanced mechanism. Conducting 30 independent experiments on CEC 2017 benchmark functions, the data indicate that OMWOA, enhanced by both mechanisms, has a significant advantage over WOA improved by either mechanism alone. Specifically, OMWOA outperforms OWOA in 8 functions and MWOA in 18 functions, demonstrating that the combined use of both mechanisms greatly enhances WOA, which is not achievable by either mechanism alone.

**Table 3:** Various WOAs with three strategies.

Algorithm	OWOA	MWOA
OMWOA	1	1
OWOA	1	0
MWOA	0	1
WOA	0	0

**Table 4:** Ablation analysis.

Algorithm	Rank	+/ = /-	AVG
OMWOA	1	~	1.8
OWOA	2	8/3/19	2.8
MWOA	3	18/5/7	2.5
WOA	4	15/3/12	2.9

Based on the ranking outcomes, it is clear that OMWOA achieves faster convergence and superior precision compared to RWOA across single-peak functions, hybrid functions, and composite functions. Additionally, OMWOA demonstrates higher precision than DWOA in multi-module benchmark functions. These results validate OMWOA as the optimal solution for improving WOA’s performance in handling these test functions. Hence, OMWOA is chosen as the preferred enhancement method for WOA following this analysis.

### 4.3 Scalability analysis

This section evaluates OMWOA’s scalability by testing it under different dimensions. Scalability tests are crucial for evaluating the performance of evolutionary computing algorithms in handling large-scale issues. By varying the problem size across various dimensions, the algorithm’s capacity to tackle different sizes and complexities can be assessed. These experiments measure the algorithm’s performance in terms of resource efficiency, time consumption, and solution quality, thereby determining its applicability and limitations. Scalability experiments are critical for providing reliable solutions to extensive problems in practical scenarios, thus promoting the wider application of evolutionary computing. The study considers three dimensions: 30, 50, and 100, which are standard benchmarks in evolutionary computing, demonstrating OMWOA’s optimization capabilities. Setting the problem dimension to 30 is a common practice in the field of evolutionary computation. When the dimension is increased to 100, the problem becomes a high-dimensional optimization task. Using a dimension of 100 better highlights the performance stability of the proposed OMWOA when addressing problems of varying difficulty. Table 5 presents the scalability analysis results, showing that OMWOA consistently outperforms WOA across all tested dimensions. Scalability tests also compare the original WOA, highlighting OMWOA’s superior performance in various dimensions.

**Table 5:** Scalability tests in three dimensions.

	Dim	30		50		100	
	Metric	OMWOA	WOA	OMWOA	WOA	OMWOA	WOA
**F1**	AVG	4.83E + 03	5.63E + 03	5.95E + 03	6.87E + 03	7.21E + 03	1.07E + 04
	STD	1.11E + 06	6.59E + 05	2.36E + 06	7.81E + 05	5.07E + 06	1.35E + 06
**F2**	AVG	2.89E + 09	1.33E + 10	7.21E + 27	3.94E + 28	7.16E + 77	3.92E + 78
	STD	2.62E + 13	6.58E + 13	8.45E + 30	4.63E + 31	1.15E + 98	6.31E + 98
**F3**	AVG	4.80E + 03	2.76E + 03	4.92E + 04	1.25E + 04	2.40E + 05	3.32E + 04
	STD	1.86E + 03	2.46E + 03	1.03E + 04	7.91E + 03	8.04E + 04	2.08E + 04
**F4**	AVG	4.71E + 02	2.77E + 01	5.05E + 02	5.41E + 01	6.41E + 02	2.91E + 01
	STD	4.99E + 02	2.00E + 01	5.59E + 02	4.93E + 01	6.53E + 02	3.44E + 01
**F5**	AVG	5.43E + 02	2.30E + 01	6.40E + 02	9.21E + 01	1.21E + 03	1.36E + 02
	STD	5.34E + 02	1.04E + 01	5.76E + 02	3.51E + 01	1.13E + 03	6.26E + 01
**F6**	AVG	6.00E + 02	7.81E-03	6.00E + 02	9.19E-03	6.00E + 02	1.17E-02
	STD	6.00E + 02	6.80E-02	6.00E + 02	2.10E-01	6.00E + 02	6.83E-02
**F7**	AVG	7.96E + 02	4.41E + 01	1.01E + 03	7.56E + 01	1.55E + 03	1.32E + 02
	STD	7.79E + 02	2.52E + 01	9.29E + 02	9.44E + 01	1.49E + 03	7.74E + 01
**F8**	AVG	8.42E + 02	9.72E + 00	9.34E + 02	8.00E + 01	1.50E + 03	1.55E + 02
	STD	8.40E + 02	1.09E + 01	9.13E + 02	7.35E + 01	1.40E + 03	1.32E + 02
**F9**	AVG	9.03E + 02	3.65E + 00	9.06E + 02	5.21E + 00	9.09E + 02	5.38E + 00
	STD	9.49E + 02	8.66E + 01	9.80E + 02	8.32E + 01	1.09E + 03	9.25E + 01
**F10**	AVG	4.21E + 03	1.34E + 03	9.19E + 03	2.73E + 03	2.61E + 04	5.68E + 03
	STD	2.96E + 03	5.30E + 02	6.29E + 03	2.46E + 03	2.36E + 04	2.04E + 03
**F11**	AVG	1.13E + 03	2.58E + 01	1.16E + 03	2.74E + 01	1.64E + 03	1.84E + 02
	STD	1.64E + 03	1.46E + 03	1.23E + 03	9.66E + 01	2.43E + 03	9.85E + 02
**F12**	AVG	1.88E + 05	1.19E + 05	1.76E + 06	1.02E + 06	2.69E + 06	1.08E + 06
	STD	2.22E + 06	1.54E + 06	9.99E + 06	3.73E + 06	2.80E + 07	1.14E + 07
**F13**	AVG	1.75E + 04	1.73E + 04	6.90E + 03	7.44E + 03	5.45E + 03	4.58E + 03
	STD	1.46E + 06	2.00E + 06	2.38E + 06	1.10E + 06	4.03E + 05	2.69E + 05
**F14**	AVG	4.95E + 04	3.94E + 04	8.64E + 04	6.27E + 04	2.70E + 05	9.63E + 04
	STD	1.05E + 06	1.01E + 06	2.31E + 06	1.79E + 06	3.39E + 06	1.72E + 06
**F15**	AVG	1.02E + 04	8.30E + 03	9.93E + 03	6.49E + 03	4.12E + 03	3.62E + 03
	STD	8.27E + 04	1.97E + 05	2.17E + 05	2.67E + 05	2.79E + 05	3.05E + 05
**F16**	AVG	2.33E + 03	3.18E + 02	2.87E + 03	4.11E + 02	6.14E + 03	2.02E + 03
	STD	2.33E + 03	2.46E + 02	2.73E + 03	3.79E + 02	4.93E + 03	1.84E + 03
**F17**	AVG	2.07E + 03	1.90E + 02	2.60E + 03	3.09E + 02	5.31E + 03	9.18E + 02
	STD	2.02E + 03	2.37E + 02	2.41E + 03	2.92E + 02	4.36E + 03	1.11E + 03
**F18**	AVG	2.43E + 05	2.36E + 05	1.27E + 06	1.18E + 06	2.79E + 06	1.54E + 06
	STD	1.41E + 06	1.99E + 06	3.86E + 06	6.13E + 06	3.21E + 06	1.98E + 06
**F19**	AVG	1.33E + 04	1.37E + 04	1.59E + 04	1.10E + 04	5.15E + 03	2.85E + 03
	STD	1.17E + 05	1.62E + 05	8.54E + 04	1.07E + 05	3.11E + 05	2.17E + 05
**F20**	AVG	2.35E + 03	1.87E + 02	2.73E + 03	2.79E + 02	5.62E + 03	1.01E + 03
	STD	2.43E + 03	2.19E + 02	2.52E + 03	2.39E + 02	4.54E + 03	9.98E + 02
**F21**	AVG	2.34E + 03	9.39E + 00	2.46E + 03	8.72E + 01	3.02E + 03	1.21E + 02
	STD	2.34E + 03	1.45E + 01	2.40E + 03	6.93E + 01	2.98E + 03	4.44E + 01
**F22**	AVG	2.30E + 03	9.48E-01	1.04E + 04	3.28E + 03	2.81E + 04	9.27E + 03
	STD	3.24E + 03	1.15E + 03	8.63E + 03	3.29E + 03	2.39E + 04	6.22E + 03
**F23**	AVG	2.71E + 03	1.73E + 01	2.83E + 03	2.13E + 01	3.03E + 03	3.21E + 01
	STD	2.71E + 03	1.57E + 01	2.82E + 03	3.02E + 01	3.06E + 03	4.69E + 01
**F24**	AVG	2.88E + 03	3.52E + 01	3.07E + 03	1.01E + 02	3.56E + 03	6.69E + 01
	STD	2.88E + 03	2.02E + 01	3.10E + 03	8.04E + 01	3.61E + 03	5.65E + 01
**F25**	AVG	2.89E + 03	9.42E + 00	3.02E + 03	3.63E + 01	3.24E + 03	5.97E + 01
	STD	2.89E + 03	1.24E + 01	3.05E + 03	3.18E + 01	3.27E + 03	4.96E + 01
**F26**	AVG	4.15E + 03	1.61E + 02	4.83E + 03	4.63E + 02	9.67E + 03	2.09E + 03
	STD	4.01E + 03	4.79E + 02	4.60E + 03	6.39E + 02	8.37E + 03	6.67E + 02
**F27**	AVG	3.23E + 03	1.65E + 01	3.39E + 03	7.04E + 01	3.48E + 03	6.10E + 01
	STD	3.24E + 03	1.83E + 01	3.46E + 03	1.02E + 02	3.55E + 03	6.83E + 01
**F28**	AVG	3.16E + 03	5.98E + 01	3.29E + 03	2.15E + 01	3.36E + 03	2.45E + 01
	STD	3.22E + 03	1.52E + 01	3.31E + 03	2.25E + 01	3.42E + 03	3.53E + 01
**F29**	AVG	3.64E + 03	1.99E + 02	3.84E + 03	3.52E + 02	5.50E + 03	5.38E + 02
	STD	3.62E + 03	1.67E + 02	3.84E + 03	2.57E + 02	4.97E + 03	3.29E + 02
**F30**	AVG	1.01E + 04	3.38E + 03	1.07E + 06	3.36E + 05	1.26E + 04	6.41E + 03
	STD	1.26E + 05	1.18E + 05	1.23E + 06	3.45E + 05	1.25E + 06	6.60E + 05
**+/-/=**	~	~	17/6/7	~	14/8/8	~	15/10/5

It is well recognized that solving problems becomes increasingly complex and challenging as the dimensions of test functions grow. Based on the above analysis, OMWOA emerges as a superior method for optimizing high-dimensional functions compared to the original WOA.

**Fig 1:**
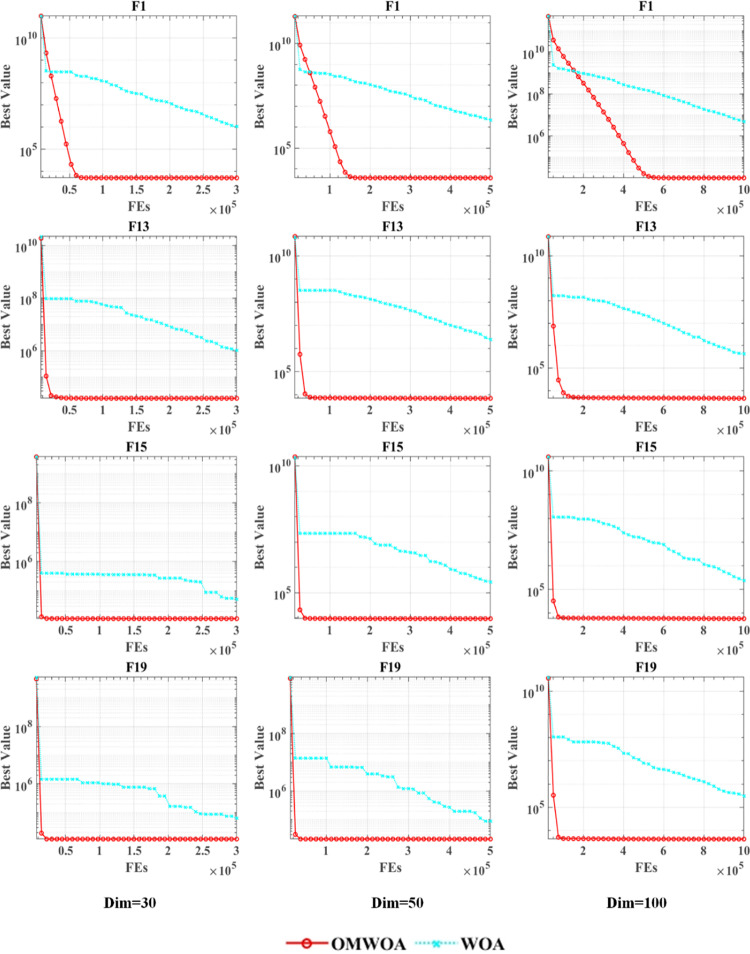
Scalability analysis on the IEEE CEC 2017 benchmark functions.

As depicted in Fig 1, the convergence trajectories of OMWOA (red) and WOA (blue) are shown for various test functions. The dimensions analyzed are 30, 50, and 100, and the chosen test functions include F1, F13, F15, and F19 from the CEC 2017 benchmark suite. Fig 1 highlights that OMWOA exhibits a faster convergence rate and higher accuracy compared to WOA.

### 4.4 Historical searches

**Fig 2:**
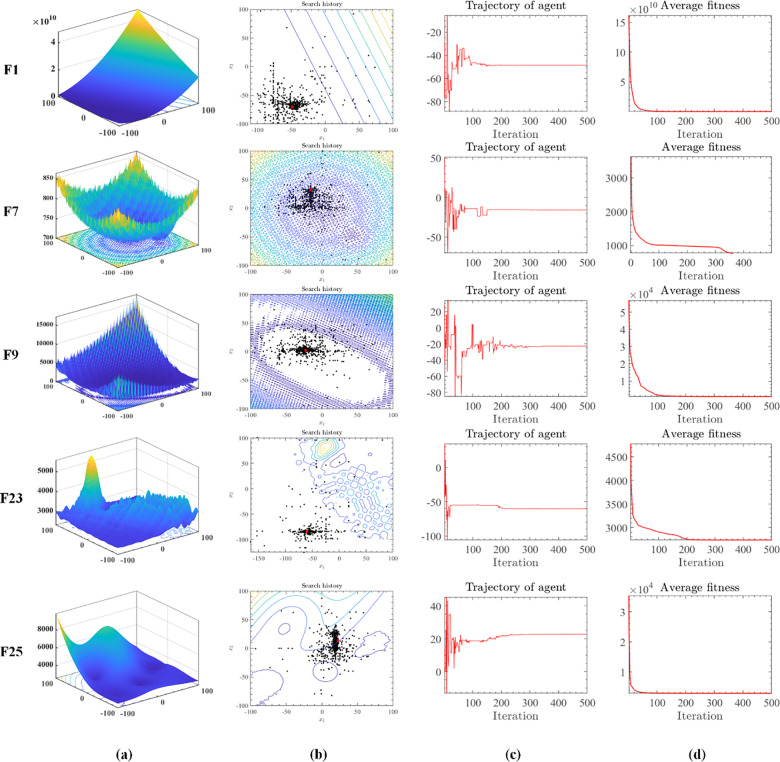
Evolutionary trajectory of OMWOA on the IEEE CEC 2017 benchmark functions.

Visualizing algorithmic search processes is critical in evolutionary computing research. Visual tools enable researchers to intuitively monitor the trajectory of the algorithm’s search within the solution space, its speed, and its ability to avoid local optima. This enhances the understanding of the algorithm’s operational principles and behavior, offering valuable insights for further optimization. Visual experiments also help identify algorithmic limitations and potential issues, guiding improvements. Therefore, visual experiments of algorithmic search processes are essential for the thorough investigation and refinement of evolutionary computing algorithms, promoting their development and practical application. To illustrate OMWOA’s search process, Fig 2 presents its historical trajectory on IEEE CEC 2017 benchmark functions, including F1, F7, F9, F23, and F25. Fig 2(a) depicts simulated images of these functions, while Fig 2(b) details the historical search path of OMWOA. Red points represent global optima, while black points indicate the optimizer’s findings at each iteration. OMWOA’s trajectory demonstrates a strong inclination towards optimal values, effectively avoiding local optima. The black dots surrounding the red dot and others evenly distributed throughout the search space highlight OMWOA’s global exploration ability. Fig 2(c) illustrates the relative discrepancy from the optimal value at each iteration, showing OMWOA’s stabilization around 500 iterations. Finally, Fig 2(d) portrays the average fitness values obtained at the conclusion of each iteration, showing an overall decreasing trend.

### 4.5 Comparison of other related algorithms

#### 4.5.1 Comparative Experiments at CEC 2017 benchmark functions.

This section evaluates OMWOA using the IEEE CEC 2017 benchmark functions. The Wilcoxon signed-rank test [37] and Friedman test [38] was employed to evaluate performance.

Table 6 presents a comprehensive comparison of OMWOA with alternative competing algorithms using the IEEE CEC 2017 benchmark functions. The competing algorithms involved in this experiment include HGWO [39], WEMFO [40], mSCA [41], SCADE [42], CCMWOA [43], QCSCA [44], BWOA [45], CCMSCSA [46], CLACO [47], BLPSO [48], GCHHO [49]. This analysis includes each algorithm’s rank, performance against OMWOA indicated by wins/draws/losses (+/ = /-), and the average performance score (AVG) across 30 independent runs. OMWOA achieves the top rank with an impressive average score of 1.30E + 00, denoted by the “~” symbol in the + / = /- column, indicating that OMWOA serves as the benchmark algorithm in this comparative study. This underscores OMWOA’s robust optimization capabilities and its ability to consistently achieve optimal solutions across the diverse set of benchmark functions. Among the competing algorithms, HGWO, despite ranking 9th, exhibits a relatively higher average score of 8.40E + 00. The 30/0/0 + / = /- metric highlights that OMWOA outperforms HGWO in all benchmark instances, emphasizing OMWOA’s superior optimization efficiency compared to HGWO. WEMFO and BLPSO, ranked 4th and 5th respectively, demonstrate competitive performance with average scores of 4.50E + 00 and 5.33E + 00. WEMFO shows a 28/0/2 + / = /- metric, indicating occasional instances where it performs better than OMWOA, while BLPSO exhibits a 25/4/1 + / = /- metric, suggesting its capability to occasionally match OMWOA’s performance. However, OMWOA maintains its superior ranking due to its overall lower average score. In contrast, algorithms like mSCA and SCADE, ranked 12th and 11th respectively, exhibit poorer performance with average scores exceeding 1.10E + 01 and no wins against OMWOA. These results underscore their limited effectiveness compared to OMWOA in achieving optimal solutions for the benchmark functions. The CCMWOA and CLACO, ranked 7th and 3rd respectively, present strong competition with average scores of 7.63E + 00 and 4.33E + 00. Despite OMWOA’s top ranking, CCMWOA achieves 29 wins against OMWOA, indicating its competitive performance in optimization scenarios. CLACO also demonstrates 26 wins against OMWOA, reinforcing its capability to outperform OMWOA in specific instances. Other algorithms such as QCSCA, BWOA, and GCHHO, ranked 6th, 8th, and 10th respectively, demonstrate mixed performance with average scores of 5.47E + 00, 8.23E + 00, and 8.53E + 00. Their + / = /- metrics indicate varying levels of competitiveness against OMWOA, with QCSCA showing occasional superiority in specific instances but generally falling short in overall performance compared to OMWOA.

**Table 6:** Experiments comparing OMWOA with alternative competing algorithms on the IEEE CEC 2017 benchmark functions.

Algorithm	Rank	+/ = /-	AVG
OMWOA	1	~	1.30E + 00
HGWO	9	30/0/0	8.40E + 00
WEMFO	4	28/0/2	4.50E + 00
mSCA	12	30/0/0	1.10E + 01
SCADE	11	30/0/0	1.04E + 01
CCMWOA	7	29/0/1	7.63E + 00
QCSCA	6	27/0/3	5.47E + 00
BWOA	8	30/0/0	8.23E + 00
CCMSCSA	2	22/0/8	2.90E + 00
CLACO	3	26/0/4	4.33E + 00
BLPSO	5	25/4/1	5.33E + 00
GCHHO	10	29/0/1	8.53E + 00

In summary, the experimental results conclusively demonstrate that OMWOA outperforms all other competing algorithms on the IEEE CEC 2017 benchmark functions. OMWOA’s top rank and the lowest average performance score underscore its effectiveness and robustness in addressing complex optimization challenges. These findings establish OMWOA as a leading optimization framework with substantial potential for practical applications across various domains.

**Fig 3:**
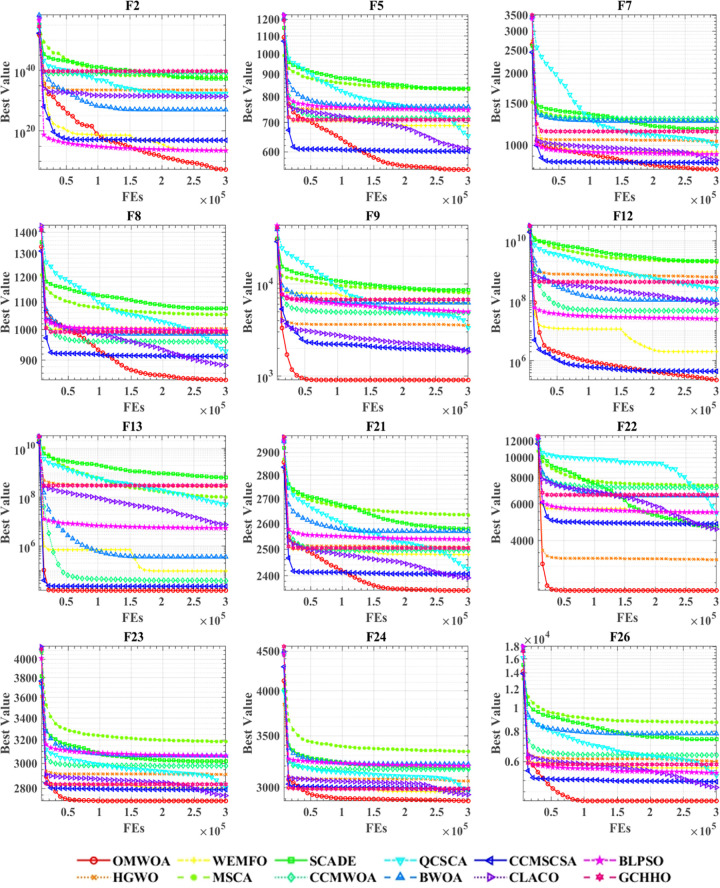
Performance comparisons of OMWOA with state-of-the-art competitors on the IEEE CEC 2017.

Understanding the convergence rate is critical for evaluating how effectively evolutionary algorithms perform and exploring their capacity for development. Fig 3 illustrates the convergence curves of OMWOA and its competitors on the CEC 2017 benchmark functions. Convergence curves are vital analytical tools in evolutionary algorithm research. These plots provide a visual summary of the convergence behavior of algorithms during optimization, displaying the progress of the search process within the solution space. By examining these curves, researchers can gain insights into the convergence speed, stability, and potential issues such as premature convergence or oscillation. Moreover, convergence curves allow for the fine-tuning of algorithm parameters to achieve optimal performance, ensuring better adaptation to specific problem-solving requirements. Therefore, these plots are crucial in evolutionary algorithm research, serving as key indicators for evaluating performance and refining algorithm designs. The graph illustrates the convergence curves for all compared algorithms across twelve test functions, with the x-axis indicating the number of iterations and the y-axis representing the optimization value. For functions F5, F8, F22, and F26, OMWOA demonstrates significant convergence advantages, quickly reaching optimal values and achieving the lowest optimal values. Even in other plots, especially in complex scenarios, where the convergence curves of various algorithms are closely clustered, OMWOA consistently achieves the best optimization values.

#### 4.5.2 Comparative Experiments at CEC 2022 benchmark functions.

The competing algorithms involved in this experiment include HGWO [39], WEMFO [40], mSCA [41], SCADE [42], CCMWOA [43], QCSCA [44], BWOA [45], CCMSCSA [46], CLACO [47], BLPSO [48], GCHHO [49]. Table 7 presents a comparative analysis of OMWOA against several competing algorithms using the IEEE CEC 2022 benchmark functions. This table provides insights into the rankings, performance distribution (+/ = /-), and average performance scores (AVG) across multiple experimental runs. “+” indicates that OMWOA outperforms the optimizer, “-” means OMWOA underperforms compared to the optimizer, and “=” denotes no significant difference in performance between OMWOA and the optimizer. The Wilcoxon signed-rank test [37] and Friedman test [38] was employed to evaluate performance. OMWOA secures the top rank denoted by the symbol “~” in the + / = /- column, indicating its superior performance compared to all other algorithms evaluated in this study. OMWOA achieves an impressive average score of 1.35E + 00, highlighting its robustness and effectiveness across the diverse and complex optimization challenges presented by the IEEE CEC 2022 benchmark functions. QCSCA follows closely with the 2nd rank, achieving an average score of 2.78E + 00 and a + / = /- metric of 6/0/6. While QCSCA performs well, it falls short of OMWOA’s top performance, indicating that OMWOA consistently achieves better average results across the benchmark functions. GCHHO and CLACO secure the 4th and 3rd ranks respectively, with average scores of 4.25E + 00 and 3.96E + 00. Their + / = /- metrics of 7/2/3 and 4/2/6 illustrate their varying degrees of competitiveness relative to OMWOA, showing instances where these algorithms perform well but do not consistently match OMWOA’s top-tier performance. HGWO occupies the 9th rank with an average score of 7.89E + 00 and a + / = /- metric of 10/1/1, indicating its lower effectiveness compared to OMWOA across the benchmark functions. SCADE, CCMWOA, BWOA, CCMSCSA, and BLPSO are positioned in the lower ranks (from 10th to 12th), demonstrating their comparative weaknesses in achieving competitive scores compared to OMWOA. In summary, OMWOA emerges as the top-performing algorithm in this comparative evaluation on the IEEE CEC 2022 benchmark functions. Its consistent top rank and superior average performance underscore OMWOA’s effectiveness and robustness in solving complex optimization problems across various domains. These findings position OMWOA as a promising choice for practitioners and researchers seeking reliable solutions in global optimization tasks.

**Table 7:** Experiments comparing OMWOA with alternative competing algorithms at IEEE CEC 2022 benchmark functions.

Algorithm	Rank	+/ = /-	AVG
OMWOA	1	~	1.35E + 00
WEMFO	6	9/2/1	5.13E + 00
mSCA	7	10/0/2	5.98E + 00
SCADE	12	10/2/0	8.74E + 00
HGWO	9	10/1/1	7.89E + 00
CCMWOA	11	9/3/0	8.03E + 00
BWOA	8	9/2/1	7.17E + 00
CCMSCSA	10	10/1/1	8.15E + 00
CLACO	3	4/2/6	3.96E + 00
QCSCA	2	6/0/6	2.78E + 00
BLPSO	5	8/2/2	4.78E + 00
GCHHO	4	7/2/3	4.25E + 00

**Fig 4:**
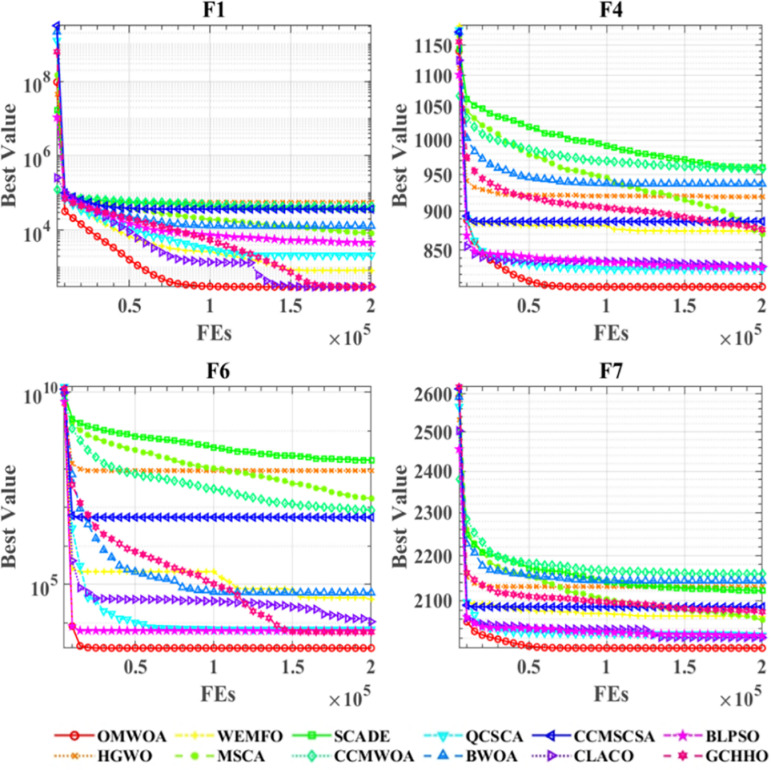
Performance comparisons of OMWOA with state-of-the-art competitors on the IEEE CEC 2022 benchmark functions.

Fig 4 illustrates the convergence curves of OMWOA and its competitors on the CEC 2022 benchmark functions. The diagram depicts the convergence behavior of all compared algorithms across nine test functions, with the x-axis indicating the number of iterations and the y-axis representing the optimization value. For functions F1, F4, F6, and F7, OMWOA shows considerable convergence advantages, swiftly reaching optimal values and achieving the lowest optimization levels. Even in other plots, particularly in complex scenarios where the convergence curves are closely clustered, OMWOA consistently attains the best optimization results.

## 5. Application to medical data analysis

Intelligent optimization algorithms play a pivotal role in addressing real-world challenges[50]. This section introduces the integration of OMWOA with KELM, a renowned machine learning model. By optimizing the two kernel parameters of KELM using OMWOA, a novel model, OMWOA-KELM, is proposed. The performance of OMWOA-KELM is evaluated against four established classifiers. The experimental data comprise real medical datasets sourced from the UCI Machine Learning Repository[51] and the Group of Applied Research in Orthopedics (GARO) at the Centre Médico-Chirurgical de Réadaptation des Massues in Lyon, France. These datasets encompass a variety of diseases, including heart disease.

The Kernel Extreme Learning Machine (KELM) was selected as the classifier in this study due to its superior learning speed, strong generalization ability, and robustness against local minima, which are critical for handling complex and high-dimensional medical data. Compared to traditional neural networks and support vector machines (SVM), KELM offers significantly faster training by avoiding iterative parameter tuning, as it analytically determines the output weights. Furthermore, by incorporating kernel functions, KELM effectively captures nonlinear relationships within the data without the need for manually designing complex network architectures. Previous studies have demonstrated that KELM often achieves competitive or even superior classification accuracy with considerably lower computational cost, making it highly suitable for real-world medical applications where both predictive performance and efficiency are essential. Therefore, integrating KELM with the proposed optimization algorithm enables a more efficient and effective classification framework.

To ensure a fair comparison and minimize the impact of parameter variability on model performance, the parameter configurations for OMWOA-KELM and WOA-KELM were kept nearly identical in this study. Specifically, the population size and maximum number of iterations were set to 10 and 50, respectively, to balance computational efficiency with adequate search capability. The hyperparameters C and γ were both constrained within the range [$2^{-5}$, $2^{5}$], which has been widely adopted in previous studies to achieve a reasonable trade-off between model complexity and generalization performance. Support Vector Machine (SVM) was employed as the baseline model, with its C and γ parameters tuned within the same range using a grid search strategy to ensure consistency across comparative evaluations. A Gaussian kernel was selected due to its proven effectiveness in handling non-linear classification tasks, and LIBSVM [52] was utilized for SVM implementation.

For the K-Nearest Neighbors (KNN) model, the number of nearest neighbors was set to 1, and the Euclidean distance metric was adopted to maintain simplicity while capturing local data structures. The Classification and Regression Tree (CART) model was configured using default parameters, as these settings have demonstrated stable performance in similar classification tasks. The Backpropagation (BP) neural network was implemented using MATLAB’s Levenberg–Marquardt algorithm, with 8 hidden neurons and a mean squared error (MSE) threshold of 0.001, which provides a balance between convergence speed and model accuracy.

Since the raw medical data could not be directly processed by the aforementioned models, data preprocessing was performed to enhance model stability and comparability. Specifically, standardization and normalization were applied to scale all features within the range of [−1, 1]. This preprocessing step ensured consistent input distributions across all models while highlighting the advantages of the proposed OMWOA-KELM algorithm when handling varying data complexities.

The standardized data were subjected to 10-fold cross-validation. This approach splits the dataset into ten subsets, using nine for training and the remaining one for validation in each iteration. Such a method maximizes the utility of limited data and ensures robust model evaluation.

### 5.1 Metrics of the classification performance

In this study, to demonstrate the exceptional performance of the proposed OMWOA-KELM in diagnosing and classifying actual medical data, we employ sensitivity, specificity, accuracy (ACC), and the Matthews correlation coefficient (MCC) as performance metrics. Their formal definitions are as follows: FN refers to false negatives, FP to false positives, TP to true positives, and TN to true negatives.


$$ACC=\frac{TP+TN}{TP+FP+FN+TN}\times 100\;\%$$ (18)

$$Sensitivity=\frac{TP}{TP+FN}\times 100\;\%$$ (19)

$$Specificity=\frac{TN}{FP+TN}\times 100\;\%$$ (20)

$$MCC=\frac{TP\times TN-FP\times FN}{\sqrt{\left(TP+FP)\times (TP+FN)\times (TN+FP)\times (TN+FN\right)}}\times 100\;\%$$ (21)

Among these four-evaluation metrics, ACC measures classification accuracy by focusing on TP and TN. Sensitivity captures the model’s adaptability and emphasizes FP and TP. Specificity is linked primarily to TN, while MCC evaluates the model’s reliability. A higher MCC indicates greater reliability.

### 5.2 Breast cancer dataset

The Breast Cancer dataset is a commonly used medical dataset proposed by Dr. Wolberg [53]. The number of instances in the dataset is 699, each with 10 attributes. Table 8 shows the results for each time after the tenfold crossover. The data in the table show that the experimental results of OMWOA -KELM are as follows: ACC is 96.57%, Sen is 96.70%, Spe is 96.02%, MCC is 92.36%.

**Table 8:** Comparison results on the Breast cancer dataset.

Models	Indicator	Mean	std	1	2	3	4	5	6	7	8	9	10
KNN	ACC	0.9542	0.0211	0.9571	0.9429	0.9714	0.9571	0.9143	0.9714	0.9565	0.9286	0.9571	0.9857
CART		0.9428	0.0309	0.9286	0.8857	0.9571	0.9571	0.9286	0.9857	0.9275	0.9714	0.9143	0.9714
BP		0.9313	0.0471	0.9429	0.9143	0.9571	0.9571	0.8857	0.9857	0.9130	0.9714	0.8286	0.9571
OMWOA -KELM		0.9657	0.0226	0.9286	0.9286	0.9714	0.9714	0.9714	1.0000	0.9565	0.9714	0.9714	0.9857
WOA-KELM		0.9599	0.0231	0.9429	0.9429	1.0000	0.9571	0.9571	0.9857	0.9565	0.9286	0.9429	0.9857
SVM		0.9356	0.0809	0.9286	0.9429	0.9714	0.7143	0.9571	1.0000	0.9420	0.9714	0.9429	0.9857
KNN	Sensitivity	0.9664	0.0295	0.9800	0.9091	0.9815	0.9535	0.9474	0.9778	0.9348	1.0000	1.0000	0.9804
CART		0.9558	0.0340	0.9600	0.8864	0.9630	0.9302	0.9474	1.0000	0.9348	1.0000	0.9756	0.9608
BP		0.9604	0.0350	0.9600	0.8864	0.9630	0.9302	0.9737	1.0000	0.9348	1.0000	0.9756	0.9804
OMWOA -KELM		0.9670	0.0326	0.9600	0.8864	0.9815	0.9535	0.9737	1.0000	0.9565	0.9783	1.0000	0.9804
WOA-KELM		0.9705	0.0326	0.9800	0.9091	1.0000	0.9535	0.9474	1.0000	0.9348	1.0000	1.0000	0.9804
SVM		0.9575	0.0575	0.9600	0.9091	0.9815	0.8140	0.9737	1.0000	0.9565	1.0000	1.0000	0.9804
KNN	Specificity	0.9324	0.0674	0.9000	1.0000	0.9375	0.9630	0.8750	0.9600	1.0000	0.7917	0.8966	1.0000
CART		0.9196	0.0574	0.8500	0.8846	0.9375	1.0000	0.9063	0.9600	0.9130	0.9167	0.8276	1.0000
BP		0.8842	0.1104	0.9000	0.9615	0.9375	1.0000	0.7813	0.9600	0.8696	0.9167	0.6207	0.8947
OMWOA -KELM		0.9602	0.0471	0.8500	1.0000	0.9375	1.0000	0.9688	1.0000	0.9565	0.9583	0.9310	1.0000
WOA-KELM		0.9395	0.0763	0.8500	1.0000	1.0000	0.9630	0.9688	0.9600	1.0000	0.7917	0.8621	1.0000
SVM		0.8972	0.1316	0.8500	1.0000	0.9375	0.5556	0.9375	1.0000	0.9130	0.9167	0.8621	1.0000
KNN	MCC	0.9010	0.0406	0.8940	0.8876	0.9190	0.9106	0.8278	0.9378	0.9094	0.8450	0.9140	0.9651
CART		0.8740	0.0646	0.8229	0.7604	0.8818	0.9150	0.8561	0.9691	0.8391	0.9373	0.8257	0.9324
BP		0.8525	0.0896	0.8600	0.8279	0.8818	0.9150	0.7783	0.9691	0.8043	0.9373	0.6605	0.8904
OMWOA -KELM		0.9236	0.0507	0.8229	0.8622	0.9190	0.9422	0.9424	1.0000	0.9037	0.9366	0.9422	0.9651
WOA-KELM		0.9145	0.0499	0.8579	0.8876	1.0000	0.9106	0.9142	0.9691	0.9094	0.8450	0.8863	0.9651
SVM		0.8584	0.1743	0.8229	0.8876	0.9190	0.3829	0.9138	1.0000	0.8696	0.9373	0.8863	0.9651

**Fig 5:**
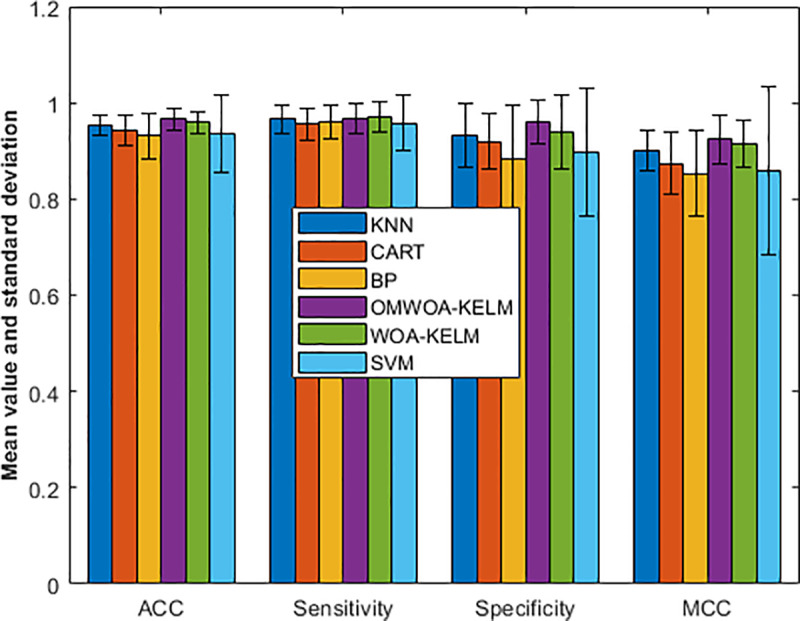
Comparison results on the Breast Cancer dataset.

In Fig 5, we graphically illustrate the experimental results, which allow us to observe the results more visually. The experimental data provides a comprehensive comparison of different models based on four evaluation metrics: accuracy (ACC), sensitivity, specificity, and Matthew’s correlation coefficient (MCC). The results include the mean and standard deviation for each metric.

For ACC, OMWOA-KELM achieves the highest mean value of 0.9657 with a standard deviation of 0.0226, surpassing other models such as WOA-KELM (0.9599 ± 0.0231) and KNN (0.9542 ± 0.0211). CART, BP, and SVM show slightly lower values of 0.9428 ± 0.0309, 0.9313 ± 0.0471, and 0.9356 ± 0.0809, respectively.

In terms of sensitivity, WOA-KELM demonstrates the best performance with a mean of 0.9705 ± 0.0326, followed closely by OMWOA-KELM (0.9670 ± 0.0326). KNN, BP, CART, and SVM achieve mean values of 0.9664, 0.9604, 0.9558, and 0.9575, respectively, with varying standard deviations.

For specificity, OMWOA-KELM again outperforms with a mean of 0.9602 ± 0.0471, whereas KNN and WOA-KELM achieve values of 0.9324 ± 0.0674 and 0.9395 ± 0.0763, respectively. CART, BP, and SVM demonstrate lower specificity values, particularly BP and SVM, which fall below 0.9.

Regarding MCC, OMWOA-KELM reaches the highest score of 0.9236 ± 0.0507, followed by WOA-KELM (0.9145 ± 0.0499) and KNN (0.9010 ± 0.0406). CART, BP, and SVM present lower reliability, with MCC values of 0.8740, 0.8525, and 0.8584, respectively.

### 5.3. Bupa liver dataset

The Bupa liver dataset is a commonly used medical dataset by Forsyth’s report [54]. The number of instances in the dataset is 345, each with 7 attributes. Table 9 shows the experimental results for the four indicators.

**Table 9:** Comparison results on the Bupa liver dataset.

models	Indicator	Mean	std	1	2	3	4	5	6	7	8	9	10
KNN	ACC	0.7630	0.0500	0.7037	0.7407	0.7407	0.7407	0.8889	0.7778	0.7778	0.7407	0.7407	0.7778
CART		0.7407	0.0676	0.6296	0.7407	0.7778	0.7778	0.7778	0.8148	0.8148	0.7407	0.6296	0.7037
BP		0.7593	0.1051	0.7778	0.6296	0.6296	0.7037	0.8519	0.8889	0.8519	0.8889	0.6667	0.7037
OMWOA -KELM		0.8296	0.0530	0.7778	0.7407	0.8519	0.8148	0.8519	0.9259	0.8889	0.8148	0.8148	0.8148
WOA-KELM		0.7667	0.0464	0.7778	0.7407	0.7407	0.7407	0.8889	0.7778	0.7778	0.7407	0.7407	0.7407
SVM		0.7074	0.1932	0.8148	0.7407	0.7407	0.5556	0.2222	0.8889	0.7778	0.7778	0.8519	0.7037
KNN	Sensitivity	0.7944	0.1035	0.6500	0.9091	0.6875	0.7857	0.8571	0.7500	0.7692	0.7857	0.7500	1.0000
CART		0.7796	0.0899	0.6000	0.8182	0.8125	0.8571	0.6429	0.8500	0.8462	0.7857	0.7500	0.8333
BP		0.8020	0.1207	0.7000	0.6364	0.7500	0.7143	0.8571	0.9000	0.8462	0.9286	0.6875	1.0000
OMWOA -KELM		0.8765	0.0655	0.7500	0.9091	0.8750	0.9286	0.9286	0.9500	0.8462	0.7857	0.8750	0.9167
WOA-KELM		0.7911	0.0796	0.7000	0.9091	0.6875	0.7857	0.8571	0.7500	0.7692	0.7857	0.7500	0.9167
SVM		0.7055	0.2657	0.7500	0.9091	0.6875	0.7143	0.0000	0.9000	0.6923	0.7143	0.9375	0.7500
KNN	Specificity	0.7578	0.1073	0.8571	0.6250	0.8182	0.6923	0.9231	0.8571	0.7857	0.6923	0.7273	0.6000
CART		0.6991	0.1196	0.7143	0.6875	0.7273	0.6923	0.9231	0.7143	0.7857	0.6923	0.4545	0.6000
BP		0.7281	0.1822	1.0000	0.6250	0.4545	0.6923	0.8462	0.8571	0.8571	0.8462	0.6364	0.4667
OMWOA -KELM		0.7854	0.0921	0.8571	0.6250	0.8182	0.6923	0.7692	0.8571	0.9286	0.8462	0.7273	0.7333
WOA-KELM		0.7721	0.1292	1.0000	0.6250	0.8182	0.6923	0.9231	0.8571	0.7857	0.6923	0.7273	0.6000
SVM		0.7244	0.1922	1.0000	0.6250	0.8182	0.3846	0.4615	0.8571	0.8571	0.8462	0.7273	0.6667
KNN	MCC	0.5419	0.0995	0.4448	0.5341	0.4973	0.4807	0.7802	0.5415	0.5549	0.4807	0.4719	0.6325
CART		0.4763	0.1344	0.2756	0.4973	0.5398	0.5587	0.5856	0.5416	0.6319	0.4807	0.2132	0.4382
BP		0.5252	0.2113	0.6139	0.2570	0.2132	0.4066	0.7033	0.7266	0.7033	0.7790	0.3202	0.5292
OMWOA -KELM		0.6602	0.0899	0.5415	0.5341	0.6932	0.6424	0.7096	0.8071	0.7790	0.6319	0.6128	0.6500
WOA-KELM		0.5487	0.0920	0.6139	0.5341	0.4973	0.4807	0.7802	0.5415	0.5549	0.4807	0.4719	0.5316
SVM		0.4140	0.4019	0.6614	0.5341	0.4973	0.1048	−0.6139	0.7266	0.5587	0.5635	0.6929	0.4144

**Fig 6:**
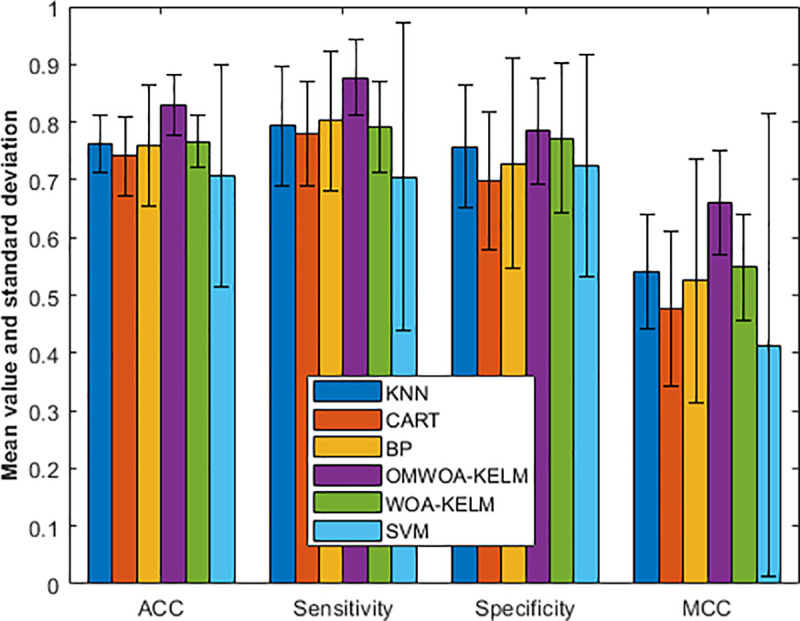
Comparison results on the Bupa liver dataset.

Specifically, we show the experimental results in Fig 6. The dataset highlights the performance of various models evaluated on four metrics: ACC, sensitivity, specificity, and MCC, reported with their mean, standard deviation, and an additional specific value.

For ACC, OMWOA-KELM achieves the highest mean value of 0.8296 ± 0.0530, outperforming models such as WOA-KELM (0.7667 ± 0.0464) and BP (0.7593 ± 0.1051). SVM demonstrates the lowest mean ACC (0.7074 ± 0.1932) despite its specific ACC reaching 0.8148, higher than most models.

In sensitivity, OMWOA-KELM again leads with a mean of 0.8765 ± 0.0655 and a specific value of 0.7500, followed by BP (0.8020 ± 0.1207). CART and SVM exhibit lower sensitivity, with respective means of 0.7796 and 0.7055, and specific values of 0.6000 and 0.7500.

Regarding specificity, BP, WOA-KELM, and SVM achieve the highest specific values (1.0000 each), although their mean values vary: BP (0.7281 ± 0.1822), WOA-KELM (0.7721 ± 0.1292), and SVM (0.7244 ± 0.1922). OMWOA-KELM follows closely, with a mean specificity of 0.7854 ± 0.0921 and a specific value of 0.8571.

For MCC, OMWOA-KELM shows the best performance with a mean of 0.6602 ± 0.0899 and a specific value of 0.5415. BP and WOA-KELM perform moderately well, with MCC means of 0.5252 and 0.5487, respectively. SVM records the lowest mean MCC (0.4140 ± 0.4019) despite a specific MCC of 0.6614.

### 5.4. Cleveland heart dataset

The Cleveland heart medical dataset is a commonly used medical dataset. The number of instances in the dataset is 303, each with 14 attributes. The results of OMWOA -KELM on the dataset are recorded in Table 10.

**Table 10:** Comparison results on the Cleveland heart dataset.

models	Indicator	Mean	std	1	2	3	4	5	6	7	8	9	10
KNN	ACC	0.7455	0.0618	0.7000	0.7333	0.7667	0.8387	0.7000	0.7097	0.7667	0.8065	0.6333	0.8000
CART		0.7553	0.1066	0.6000	0.6333	0.7333	0.8065	0.7667	0.7742	0.7667	0.8387	0.6667	0.9667
BP		0.7092	0.0929	0.8000	0.6000	0.7667	0.6452	0.5667	0.7419	0.6667	0.8387	0.6667	0.8000
OMWOA -KELM		0.8316	0.0529	0.7333	0.8667	0.8333	0.8710	0.8667	0.7742	0.8000	0.8710	0.8000	0.9000
WOA-KELM		0.7488	0.0640	0.7000	0.7333	0.7667	0.8387	0.7000	0.7097	0.8000	0.8065	0.6333	0.8000
SVM		0.8017	0.0903	0.5667	0.8000	0.8333	0.8710	0.8333	0.7419	0.8333	0.8710	0.8333	0.8333
KNN	Sensitivity	0.7099	0.0901	0.9000	0.7500	0.7778	0.6000	0.7500	0.6471	0.6429	0.7333	0.6316	0.6667
CART		0.7047	0.1159	0.6000	0.6250	0.7778	0.5000	0.7500	0.7647	0.6429	0.7333	0.7368	0.9167
BP		0.6869	0.1243	0.8000	0.6250	0.7222	0.7000	0.4375	0.7647	0.5714	0.8667	0.6316	0.7500
OMWOA -KELM		0.7722	0.0687	0.7000	0.8750	0.8333	0.8000	0.8125	0.6471	0.7143	0.8000	0.7895	0.7500
WOA-KELM		0.7171	0.0870	0.9000	0.7500	0.7778	0.6000	0.7500	0.6471	0.7143	0.7333	0.6316	0.6667
SVM		0.7757	0.1141	0.7000	1.0000	0.8333	0.8000	0.8125	0.5882	0.7143	0.8000	0.8421	0.6667
KNN	Specificity	0.7733	0.1224	0.6000	0.7273	0.7500	0.9524	0.6429	0.7857	0.8750	0.8750	0.6364	0.8889
CART		0.7785	0.1609	0.6000	0.6364	0.6667	0.9524	0.7857	0.7857	0.8750	0.9375	0.5455	1.0000
BP		0.7395	0.0848	0.8000	0.5909	0.8333	0.6190	0.7143	0.7143	0.7500	0.8125	0.7273	0.8333
OMWOA -KELM		0.8840	0.0715	0.7500	0.8636	0.8333	0.9048	0.9286	0.9286	0.8750	0.9375	0.8182	1.0000
WOA-KELM		0.7733	0.1224	0.6000	0.7273	0.7500	0.9524	0.6429	0.7857	0.8750	0.8750	0.6364	0.8889
SVM		0.8389	0.1379	0.5000	0.7273	0.8333	0.9048	0.8571	0.9286	0.9375	0.9375	0.8182	0.9444
KNN	MCC	0.4862	0.1116	0.4757	0.4308	0.5218	0.6176	0.3955	0.4328	0.5361	0.6161	0.2588	0.5774
CART		0.4923	0.2226	0.1890	0.2332	0.4444	0.5353	0.5345	0.5481	0.5361	0.6883	0.2823	0.9319
BP		0.4183	0.1788	0.5774	0.1914	0.5443	0.2984	0.1571	0.4790	0.3273	0.6792	0.3459	0.5833
OMWOA -KELM		0.6559	0.1066	0.4330	0.6929	0.6591	0.7048	0.7411	0.5881	0.6001	0.7469	0.5909	0.8018
WOA-KELM		0.4926	0.1165	0.4757	0.4308	0.5218	0.6176	0.3955	0.4328	0.6001	0.6161	0.2588	0.5774
SVM		0.6129	0.1577	0.1903	0.6447	0.6591	0.7048	0.6682	0.5375	0.6748	0.7469	0.6495	0.6533

**Fig 7:**
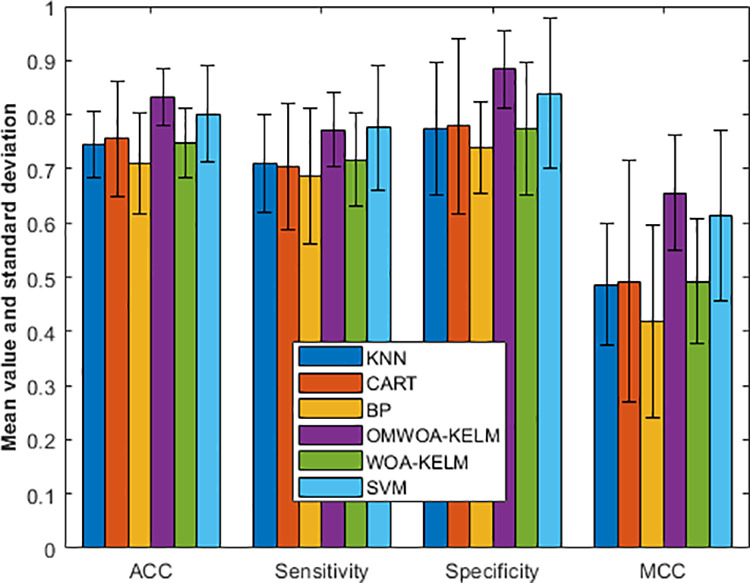
Comparison results on the Cleveland heart dataset.

Specifically, we show the experimental results in Fig 7. The dataset evaluates six models using four metrics: accuracy (ACC), sensitivity, specificity, and Matthew’s correlation coefficient (MCC), presented as mean ± standard deviation.

For ACC, OMWOA-KELM outperforms other models with a mean of 0.8316 ± 0.0529, followed by SVM (0.8017 ± 0.0903). Models such as CART (0.7553 ± 0.1066) and WOA-KELM (0.7488 ± 0.0640) show moderate accuracy, while BP scores the lowest with 0.7092 ± 0.0929.

In sensitivity, SVM and OMWOA-KELM achieve comparable results with 0.7757 ± 0.1141 and 0.7722 ± 0.0687, respectively. WOA-KELM (0.7171 ± 0.0870) and KNN (0.7099 ± 0.0901) demonstrate slightly lower sensitivity, while CART and BP perform less favorably, with means of 0.7047 ± 0.1159 and 0.6869 ± 0.1243, respectively.

For specificity, OMWOA-KELM excels with 0.8840 ± 0.0715, followed by SVM (0.8389 ± 0.1379). Other models, including CART (0.7785 ± 0.1609), KNN (0.7733 ± 0.1224), and WOA-KELM (0.7733 ± 0.1224), demonstrate moderate performance. BP shows the lowest specificity at 0.7395 ± 0.0848.

Regarding MCC, OMWOA-KELM achieves the best reliability with 0.6559 ± 0.1066. SVM follows with 0.6129 ± 0.1577, while KNN, CART, and WOA-KELM have comparable values, ranging from 0.4862 to 0.4926. BP exhibits the lowest MCC (0.4183 ± 0.1788), indicating reduced reliability.

### 5.5. Diabetes dataset

The Diabetes dataset is a commonly used medical dataset. The number of instances in the dataset is 768, each with 8 attributes. The results of OMWOA -KELM on the dataset are recorded in Table 11. The data in the table show the experimental results of OMWOA -KELM.

**Table 11:** Comparison results on the Diabetes dataset.

models	Indicator	Mean	std	1	2	3	4	5	6	7	8	9	10
KNN	ACC	0.7520	0.0645	0.7333	0.8065	0.6667	0.8333	0.7000	0.6667	0.7419	0.8000	0.7333	0.8387
CART		0.7387	0.1034	0.8667	0.8387	0.5333	0.6667	0.6667	0.6667	0.8065	0.8000	0.8000	0.7419
BP		0.7294	0.0868	0.7667	0.7097	0.6000	0.8333	0.7000	0.7667	0.6774	0.8333	0.6000	0.8065
OMWOA -KELM		0.8317	0.0287	0.8333	0.8065	0.8333	0.8333	0.8333	0.8000	0.8387	0.9000	0.8000	0.8387
WOA-KELM		0.7720	0.0812	0.7667	0.8065	0.7000	0.8333	0.7000	0.6667	0.7419	0.9333	0.7333	0.8387
SVM		0.7885	0.1172	0.4667	0.8065	0.8000	0.8333	0.7667	0.8667	0.8065	0.8333	0.8667	0.8387
KNN	Sensitivity	0.7175	0.1393	0.6667	0.8333	0.5000	0.9286	0.5556	0.6667	0.6667	0.8333	0.6667	0.8571
CART		0.7300	0.1468	0.8667	0.9444	0.4444	0.6429	0.7778	0.7500	0.6667	0.7500	0.6000	0.8571
BP		0.6903	0.1261	0.6000	0.6667	0.5556	0.8571	0.6667	0.7500	0.5833	0.8333	0.5333	0.8571
OMWOA -KELM		0.7813	0.1006	0.7333	0.7778	0.7778	0.8571	0.8889	0.6667	0.7500	0.8333	0.6000	0.9286
WOA-KELM		0.7463	0.1525	0.7333	0.8333	0.5556	0.9286	0.5556	0.6667	0.6667	1.0000	0.6667	0.8571
SVM		0.7630	0.1691	0.3333	0.7778	0.7778	0.8571	0.8889	0.8333	0.6667	0.8333	0.7333	0.9286
KNN	Specificity	0.7855	0.0627	0.8000	0.7692	0.9167	0.7500	0.7619	0.6667	0.7895	0.7778	0.8000	0.8235
CART		0.7518	0.1355	0.8667	0.6923	0.6667	0.6875	0.6190	0.6111	0.8947	0.8333	1.0000	0.6471
BP		0.7675	0.0803	0.9333	0.7692	0.6667	0.8125	0.7143	0.7778	0.7368	0.8333	0.6667	0.7647
OMWOA -KELM		0.8811	0.0723	0.9333	0.8462	0.9167	0.8125	0.8095	0.8889	0.8947	0.9444	1.0000	0.7647
WOA-KELM		0.7966	0.0705	0.8000	0.7692	0.9167	0.7500	0.7619	0.6667	0.7895	0.8889	0.8000	0.8235
SVM		0.8188	0.1088	0.6000	0.8462	0.8333	0.8125	0.7143	0.8889	0.8947	0.8333	1.0000	0.7647
KNN	MCC	0.5030	0.1334	0.4709	0.6026	0.4330	0.6832	0.3086	0.3273	0.4561	0.6001	0.4709	0.6778
CART		0.4897	0.1946	0.7333	0.6722	0.1111	0.3304	0.3637	0.3546	0.5850	0.5833	0.6547	0.5085
BP		0.4561	0.1762	0.5657	0.4304	0.2182	0.6682	0.3563	0.5218	0.3202	0.6591	0.2018	0.6193
OMWOA -KELM		0.6671	0.0552	0.6804	0.6161	0.6804	0.6682	0.6533	0.5774	0.6564	0.7907	0.6547	0.6933
WOA-KELM		0.5414	0.1727	0.5345	0.6026	0.4801	0.6832	0.3086	0.3273	0.4561	0.8729	0.4709	0.6778
SVM		0.5790	0.2366	−0.0692	0.6161	0.6001	0.6682	0.5541	0.7222	0.5850	0.6591	0.7609	0.6933

**Fig 8:**
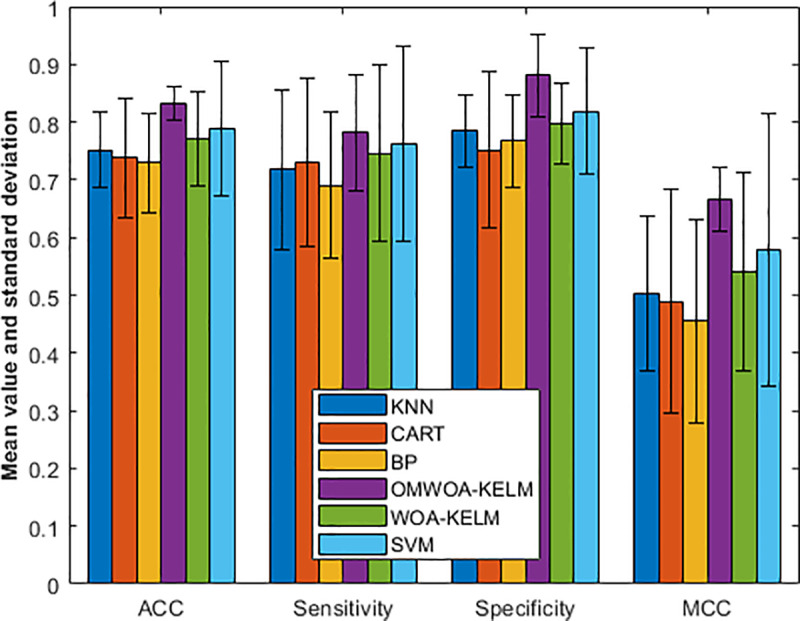
Comparison results on the Diabetes dataset.

Specifically, we show the experimental results in Fig 8. The performance of six models is evaluated across four key metrics: accuracy (ACC), sensitivity, specificity, and Matthew’s correlation coefficient (MCC), with results presented as mean ± standard deviation.

In terms of ACC, OMWOA-KELM shows the best performance, achieving 0.8317 ± 0.0287, followed by SVM at 0.7885 ± 0.1172. WOA-KELM and KNN demonstrate moderate accuracy, scoring 0.7720 ± 0.0812 and 0.7520 ± 0.0645, respectively. CART (0.7387 ± 0.1034) and BP (0.7294 ± 0.0868) yield the lowest accuracy values.

For sensitivity, OMWOA-KELM leads again with a mean of 0.7813 ± 0.1006, while SVM and WOA-KELM follow with values of 0.7630 ± 0.1691 and 0.7463 ± 0.1525, respectively. KNN and CART perform moderately, recording 0.7175 ± 0.1393 and 0.7300 ± 0.1468, while BP lags behind at 0.6903 ± 0.1261.

For specificity, OMWOA-KELM achieves the highest value of 0.8811 ± 0.0723, significantly outperforming other models. SVM follows with 0.8188 ± 0.1088, and WOA-KELM performs moderately with 0.7966 ± 0.0705. KNN (0.7855 ± 0.0627), BP (0.7675 ± 0.0803), and CART (0.7518 ± 0.1355) show relatively lower values.

In MCC, OMWOA-KELM stands out with a mean of 0.6671 ± 0.0552. SVM and WOA-KELM follow with values of 0.5790 ± 0.2366 and 0.5414 ± 0.1727, respectively. KNN, CART, and BP perform less favorably, with MCC scores ranging from 0.5030 to 0.4561.

### 5.6. Heart dataset

The Heart medical dataset is a commonly used medical dataset. The number of instances in the dataset is 270, each with 13 attributes. The results of OMWOA -KELM on the dataset are recorded in Table 12. The data in the table show the experimental results of OMWOA -KELM.

**Table 12:** Comparison results on the heart dataset.

models	Indicator	Mean	std	1	2	3	4	5	6	7	8	9	10
KNN	ACC	0.7370	0.0809	0.6667	0.7407	0.8889	0.7778	0.7407	0.6667	0.6667	0.6667	0.8519	0.7037
CART		0.7556	0.0975	0.7778	0.8519	0.8148	0.8519	0.6667	0.5926	0.6667	0.8148	0.8519	0.6667
BP		0.6630	0.1068	0.5185	0.5556	0.7778	0.7407	0.5556	0.6296	0.7037	0.6296	0.8519	0.6667
OMWOA -KELM		0.8148	0.0552	0.7778	0.8148	0.8148	0.8148	0.9259	0.7407	0.7778	0.7778	0.8889	0.8148
WOA-KELM		0.7704	0.0834	0.8519	0.7778	0.8889	0.7778	0.7407	0.6667	0.6667	0.7407	0.8889	0.7037
SVM		0.7407	0.0781	0.7407	0.7778	0.8148	0.8148	0.7778	0.7407	0.6667	0.7407	0.5556	0.7778
KNN	Sensitivity	0.7798	0.1097	0.6842	0.7500	0.9000	0.8750	0.7333	0.7500	0.6500	0.7500	1.0000	0.7059
CART		0.8007	0.1447	0.7368	0.8750	1.0000	0.8750	0.6000	0.8333	0.6000	0.9167	0.9231	0.6471
BP		0.6669	0.1344	0.5263	0.6250	0.7000	0.6875	0.6667	0.6667	0.6500	0.5000	1.0000	0.6471
OMWOA -KELM		0.8728	0.0887	0.8421	0.8125	1.0000	0.8750	0.9333	0.9167	0.7500	0.8333	1.0000	0.7647
WOA-KELM		0.8102	0.1032	0.8421	0.8125	0.9000	0.8750	0.7333	0.7500	0.6500	0.8333	1.0000	0.7059
SVM		0.7668	0.1367	0.8421	0.7500	1.0000	0.7500	0.7333	0.8333	0.7000	0.8333	0.4615	0.7647
KNN	Specificity	0.6950	0.0858	0.6250	0.7273	0.8824	0.6364	0.7500	0.6000	0.7143	0.6000	0.7143	0.7000
CART		0.7443	0.1352	0.8750	0.8182	0.7059	0.8182	0.7500	0.4000	0.8571	0.7333	0.7857	0.7000
BP		0.6618	0.1603	0.5000	0.4545	0.8235	0.8182	0.4167	0.6000	0.8571	0.7333	0.7143	0.7000
OMWOA -KELM		0.7669	0.1084	0.6250	0.8182	0.7059	0.7273	0.9167	0.6000	0.8571	0.7333	0.7857	0.9000
WOA-KELM		0.7338	0.0934	0.8750	0.7273	0.8824	0.6364	0.7500	0.6000	0.7143	0.6667	0.7857	0.7000
SVM		0.7114	0.1275	0.5000	0.8182	0.7059	0.9091	0.8333	0.6667	0.5714	0.6667	0.6429	0.8000
KNN	MCC	0.4697	0.1686	0.2874	0.4719	0.7689	0.5330	0.4807	0.3500	0.3213	0.3500	0.7391	0.3944
CART		0.5338	0.1793	0.5622	0.6932	0.6860	0.6932	0.3500	0.2539	0.4009	0.6500	0.7127	0.3354
BP		0.3235	0.2294	0.0240	0.0795	0.5235	0.4973	0.0857	0.2652	0.4448	0.2401	0.7391	0.3354
OMWOA -KELM		0.6318	0.1197	0.4671	0.6236	0.6860	0.6128	0.8500	0.5316	0.5415	0.5635	0.7990	0.6424
WOA-KELM		0.5365	0.1662	0.6781	0.5398	0.7689	0.5330	0.4807	0.3500	0.3213	0.5000	0.7990	0.3944
SVM		0.4714	0.1821	0.3565	0.5587	0.6860	0.6481	0.5635	0.5000	0.2463	0.5000	0.1062	0.5488

**Fig 9:**
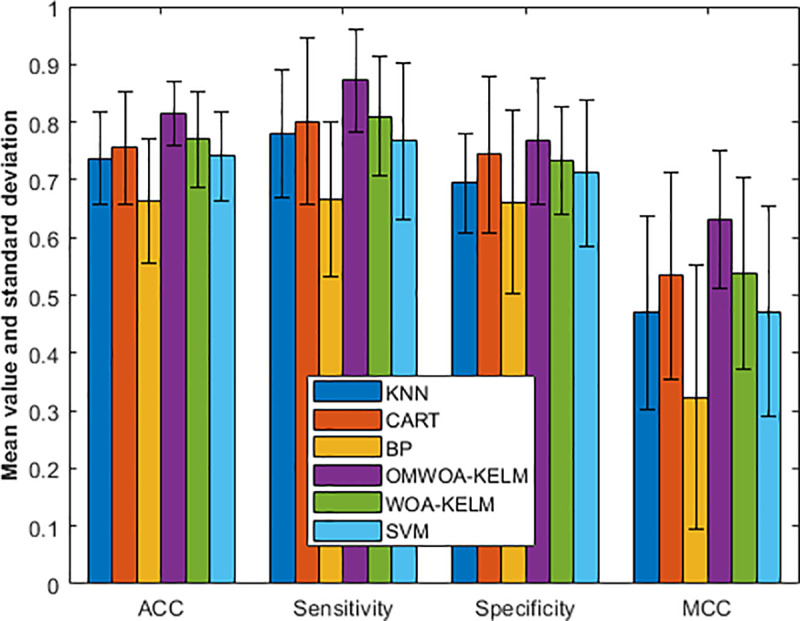
Comparison results on the Heart dataset.

Specifically, we show the experimental results in Fig 9. The models are evaluated across four key indicators. OMWOA-KELM achieves the highest performance in all metrics, particularly excelling in sensitivity (0.8728) and MCC (0.6318). CART demonstrates strong performance in specificity (0.7443) and MCC (0.5338), while SVM excels in accuracy (0.7407). WOA-KELM performs well, especially in sensitivity (0.8102), but overall, it lags behind OMWOA-KELM. KNN and BP show lower scores, especially in MCC and specificity.

## 6. Conclusions and future works

In this study, we introduced OMWOA, an enhanced version of the Whale Optimization Algorithm, which incorporates the outpost and multi-population mechanisms to address the common challenges of slow convergence and local optima stagnation. The experimental evaluation, benchmarked against prominent evolutionary algorithms from the IEEE CEC 2017 and IEEE CEC 2022 competitions, demonstrated that OMWOA outperforms traditional algorithms in terms of both convergence rate and solution quality. Furthermore, the scalability analysis showed that OMWOA retains its high performance across a wide range of problem dimensionalities. To assess its practical utility, we applied OMWOA in conjunction with KELM to solve the medical disease diagnosis problem. The results from five different medical datasets suggest that the proposed algorithm not only excels in theoretical optimization but also holds promise for real-world applications.

The proposed algorithm also has certain limitations, such as the lack of validation of its optimization performance in other real-world applications. Future work could further refine the scalability of OMWOA in more complex and high-dimensional real-world problems, and explore its integration with other machine learning models for broader applicability.
